# Evaluation of an Automated System for Reading and Interpreting Disk Diffusion Antimicrobial Susceptibility Testing of Fastidious Bacteria

**DOI:** 10.1371/journal.pone.0159183

**Published:** 2016-07-08

**Authors:** Evgeny A. Idelevich, Karsten Becker, Janne Schmitz, Dennis Knaack, Georg Peters, Robin Köck

**Affiliations:** 1 Institute of Medical Microbiology, University Hospital Münster, Münster, Germany; 2 Institute of Hygiene, University Hospital Münster, Münster, Germany; University of Malaya, MALAYSIA

## Abstract

Results of disk diffusion antimicrobial susceptibility testing depend on individual visual reading of inhibition zone diameters. Therefore, automated reading using camera systems might represent a useful tool for standardization. In this study, the ADAGIO automated system (Bio-Rad) was evaluated for reading disk diffusion tests of fastidious bacteria. 144 clinical isolates (68 β-haemolytic streptococci, 28 *Streptococcus pneumoniae*, 18 viridans group streptococci, 13 *Haemophilus influenzae*, 7 *Moraxella catarrhalis*, and 10 *Campylobacter jejuni*) were tested on Mueller-Hinton agar supplemented with 5% defibrinated horse blood and 20 mg/L β-NAD (MH-F, Oxoid) according to EUCAST. Plates were read manually with a ruler and automatically using the ADAGIO system. Inhibition zone diameters, indicated by the automated system, were visually controlled and adjusted, if necessary. Among 1548 isolate-antibiotic combinations, comparison of automated vs. manual reading yielded categorical agreement (CA) without visual adjustment of the automatically determined zone diameters in 81.4%. In 20% (309 of 1548) of tests it was deemed necessary to adjust the automatically determined zone diameter after visual control. After adjustment, CA was 94.8%; very major errors (false susceptible interpretation), major errors (false resistant interpretation) and minor errors (false categorization involving intermediate result), calculated according to the ISO 20776–2 guideline, accounted to 13.7% (13 of 95 resistant results), 3.3% (47 of 1424 susceptible results) and 1.4% (21 of 1548 total results), respectively, compared to manual reading. The ADAGIO system allowed for automated reading of disk diffusion testing in fastidious bacteria and, after visual validation of the automated results, yielded good categorical agreement with manual reading.

## Introduction

Antimicrobial susceptibility testing (AST) is essential for appropriate and targeted treatment of bacterial infections [1]. Hence, correct and reproducible AST methodology is crucial [2]. Variability in performing the test itself but also in reading the results can impact on the final finding and thus should be standardized as much as possible. The European Committee on Antimicrobial Susceptibility Testing (EUCAST) has provided methodology [3] and interpretative criteria [4] for disk diffusion susceptibility testing of fastidious bacteria. Even if the testing methodology is strictly observed, variation in endpoint reading cannot be excluded and depends from the experience of the observer. Furthermore, potential mistakes in interpretation of zone inhibition diameters should be avoided by easier accessibility of interpretative criteria. Therefore, automated reading using camera systems and automated interpretation software might represent a useful tool for standardization. A possible use of automated zone readers to measure the diameters of zones of inhibition is mentioned in the EUCAST guideline [3] and such systems have previously been evaluated [5–11]. However, automatic reading of disk diffusion tests of fastidious bacteria might represent a challenge due to the growth of tiny colonies and the need to use the special Mueller-Hinton agar for fastidious microorganisms (MH-F), recommended by EUCAST [3]. Literature evaluating automated zone readers for use with MH-F agar is sparse [10]. In this study, we evaluated the ADAGIO automated system (Bio-Rad, Marnes-la-Coquette, France) for reading and interpreting disk diffusion AST results on a collection of different fastidious bacteria.

## Materials and Methods

144 clinical isolates were tested, including 68 β-haemolytic streptococci (29 *Streptococcus agalactiae*, 14 *Streptococcus dysgalactiae*, 25 *Streptococcus pyogenes*), 28 *Streptococcus pneumoniae*, 18 viridans group streptococci, 13 *Haemophilus influenzae*, 7 *Moraxella catarrhalis*, and 10 *Campylobacter jejuni*. The most recent isolates of each species (only one per patient) were included into the study. All isolates were cultured overnight before testing. AST was performed by disk diffusion according to the EUCAST guideline [3]. Briefly, a McFarland 0.5 suspension was prepared in saline from several colonies. This suspension was inoculated within 15 minutes of preparation onto Mueller-Hinton agar plates supplemented with 5% defibrinated horse blood and 20 mg/L β-NAD (MH-F, Oxoid, Wesel, Germany) using plate rotator (Type EPA, Robin SA, Beaucouzé, France). Antibiotic disks (Oxoid) were added within 15 minutes after inoculation, followed by incubation for 18±2h at 35±1°C in 5% CO_2_, except for *C*. *jejuni*. *C*. *jejuni* isolates were incubated for 24 hours at 41±1°C in microaerobic environment, created by placing CampyGen sachet (Oxoid) into sealed jars. *Campylobacter* isolates without sufficient growth after 24 h incubation were immediately reincubated and inhibition zones read after a total of 40–48 h incubation. Up to 12 antibiotics were tested for each group of microorganisms depending on the number of antibiotics for which specific EUCAST breakpoints were available (Table 1).

**Table 1:** Susceptibility of isolates determined by manual reading (standard method) and performance of automated reading of disk diffusion antimicrobial susceptibility testing (with visual adjustment) for fastidious bacteria, compared to manual reading as standard method, n = 144.

Antimicrobial agent	No. of isolate-antibiotic combinations	No. of resistant isolates^a^	No. of susceptible isolates	No. (%) of very major errors^b^	No. (%) of major errors^b^	No. (%) of minor errors^b^	Categorical agreement, %
***S*. *agalactiae*, n = 29**	**348**	**31**	**314**	**0 (0)**	**2 (0.6)**	**3 (0.9)**	**98.6**
Penicillin G	29	0	29				
Levofloxacin	29	0	29				
Moxifloxacin	29	0	29				
Norfloxacin	29	0	29				
Teicoplanin	29	0	29		1		
Vancomycin	29	0	29				
Erythromycin	29	7	20			2	
Clindamycin	29	4	25		1		
Tetracycline	29	20	8			1	
Tigecycline	29	0	29				
Linezolid	29	0	29				
Trimethoprim/ sulfamethoxazole	29	0	29				
***S*. *dysgalactiae*, n = 14**	**168**	**12**	**152**	**1 (8.3)**	**7 (4.6)**	**4 (2.4)**	**92.9**
Penicillin G	14	0	14				
Levofloxacin	14	2	12		1		
Moxifloxacin	14	0	14				
Norfloxacin	14	1	13	1			
Teicoplanin	14	0	14				
Vancomycin	14	0	14				
Erythromycin	14	2	12				
Clindamycin	14	1	13				
Tetracycline	14	6	6		2	2	
Tigecycline	14	0	13		2	1	
Linezolid	14	0	14		1		
Trimethoprim/ sulfamethoxazole	14	0	13		1	1	
***S*. *pyogenes*, n = 25**	**300**	**6**	**293**	**1 (16.7)**	**1 (0.3)**	**1 (0.3)**	**99.0**
Penicillin G	25	0	25				
Levofloxacin	25	0	25				
Moxifloxacin	25	0	25				
Norfloxacin	25	2	23	1	1		
Teicoplanin	25	0	25				
Vancomycin	25	0	25				
Erythromycin	25	0	24			1	
Clindamycin	25	0	25				
Tetracycline	25	4	21				
Tigecycline	25	0	25				
Linezolid	25	0	25				
Trimethoprim/ sulfamethoxazole	25	0	25				
***S*. *pneumoniae*, n = 28**	**336**	**29**	**301**	**4 (13.8)**	**9 (3.0)**	**3 (0.9)**	**95.2**
Levofloxacin	28	0	28				
Moxifloxacin	28	0	28				
Norfloxacin	28	1	27	1			
Teicoplanin	28	0	28		3		
Vancomycin	28	0	28		1		
Erythromycin	28	7	21	1	1		
Clindamycin	28	5	23	1			
Tetracycline	28	5	23	1	1		
Linezolid	28	0	28		2		
Trimethoprim/ sulfamethoxazole	28	4	24		1		
Oxacillin	28	7	21				
Cefaclor	28	0	22			3	
**Viridans group streptococci, n = 18**	**126**	**3**	**123**	**0 (0)**	**0 (0)**	**0 (0)**	**100**
Penicillin G	18	0	18				
Teicoplanin	18	0	18				
Vancomycin	18	0	18				
Ampicillin	18	0	18				
Cefotaxime	18	0	18				
Cefuroxime iv^c^	18	2	16				
Cefepime	18	1	17				
***H*. *influenzae*, n = 13**	**156**	**8**	**134**	**7 (87.5)**	**18 (13.4)**	**9 (5.8)**	**78.2**
Penicillin G	13	2	11	2			
Levofloxacin	13	0	13		5		
Erythromycin	13	0	0			8	
Tetracycline	13	0	13				
Trimethoprim/ sulfamethoxazole	13	1	11	1		1	
Ampicillin	13	1	12				
Amoxicillin/clavulanic acid	13	1	12	1	1		
Cefotaxime	13	0	13		1		
Cefuroxime iv^c^	13	3	10	3	2		
Meropenem	13	0	13				
Ciprofloxacin	13	0	13		2		
Nalidixic acid	13	0	13		7		
***M*. *catarrhalis*, n = 7**	**84**	**1**	**82**	**0 (0)**	**5 (6.1)**	**1 (1.2)**	**92.9**
Levofloxacin	7	0	7				
Moxifloxacin	7	0	7				
Erythromycin	7	0	7				
Tetracycline	7	0	7		1		
Trimethoprim/ sulfamethoxazole	7	0	7				
Amoxicillin/clavulanic acid	7	0	7				
Cefotaxime	7	0	7		1		
Cefuroxime iv^c^	7	0	7				
Meropenem	7	0	7		3		
Ciprofloxacin	7	0	7				
Nalidixic acid	7	1	6				
Cefixime	7	0	6			1	
***C*. *jejuni*, n = 10**	**30**	**5**	**25**	**0 (0)**	**5 (20.0)**	**0 (0)**	**83.3**
Erythromycin	10	0	10		2		
Tetracycline	10	2	8		3		
Ciprofloxacin	10	3	7				
**TOTAL, n = 144**	**1548**	**95**	**1424**	**13 (13.7)**	**47 (3.3)**	**21 (1.4)**	**94.8**

^a^ Number of results within intermediate category can be calculated by subtracting resistant and susceptible results from the number of isolate-antibiotic combinations tested

^b^ Error rates are reported as required by the ISO 20776–2 guideline: VME (%), number of VMEs (i.e. false susceptible results) divided by the number of isolates determined resistant by the standard method; ME (%), number of MEs (i.e. false resistant results) divided by the number of isolates determined susceptible by the standard method); mE (%), number of mEs (i.e. false categorization involving intermediate result) divided by the total number of tested isolates; Categorical agreement (i.e. results within the same interpretative category)

^c^ iv, interpretation according to breakpoints for intravenous use.

Plates were read manually with a ruler by two investigators which were blinded to the results of each other and those of the automated reading. Mean zone diameter was calculated and used for categorization. All plates were also automatically read using the ADAGIO system (Bio-Rad). Thereafter, inhibition zone diameters, indicated by the automated system, were visually controlled and adjusted, if necessary. The isolates were categorized as susceptible, intermediate or resistant according to the EUCAST interpretative criteria [4]. Very major errors (VME, number of false susceptible results divided by the number of isolates determined resistant by the standard method), major errors (ME, number of false resistant results divided by the number of isolates determined susceptible by the standard method) and minor errors (mE, false categorization involving intermediate result divided by the total number of tested isolates) as well as categorical agreement (CA, results within the same interpretative category) were calculated according to the ISO 20776–2 guideline [12] for automated reading, and automated reading with visual adjustment, each compared to the manual reading as standard method.

Reference strains *Streptococcus pneumoniae* ATCC 49619, *Haemophilus influenzae* NCTC 8468 and *Campylobacter jejuni* ATCC 33560 served as quality control (QC), in accordance with the EUCAST guidelines [3,4]. Inhibition zones of QC strains were found to be within acceptable limits throughout the testing.

## Results

Overall, 1548 isolate-antibiotic combinations were tested for 144 isolates. In 20.0% (309/1548) of isolate-antibiotic combinations it was deemed necessary to adjust the automatically determined zone diameter after visual control. The mean zone diameter adjustment was +9.1 mm (median + 13 mm, min -21 mm, max +35 mm). Adjustment after visual control of at least one isolate-antibiotic combination was performed in 67.4% of all isolates (97/144): 69.0% (20/29) of *S*. *agalactiae*, 92.9% (13/14) of *S*. *dysgalactiae*, 96% (24/25) of *S*. *pyogenes*, 78.6% (22/28) of *S*. *pneumoniae*, 15.4% (2/13) of *H*. *influenza*, 57.1% (4/7) of *M*. *catarrhalis*, 50% (9/18) of viridans group streptococci, 30% (3/10) of *C*. *jejuni*.

After adjustment, VME, ME, mE rates and CA occurred in 13.7% (of resistant isolates), 3.3% (of susceptible isolates), 1.4% and 94.8%, respectively, compared to manual reading as the standard method (Table 1). Without adjustment, VME, ME, mE rates and CA were 14.7% (of resistant isolates), 17.6% (of susceptible isolates), 1.5% and 81.4%, compared to manual reading, respectively (S1 Table).

If all errors are calculated based on the total number of isolates tested (in deviation from the ISO guideline [12]), the VME and ME rates would amount to 0.8% and 3.0% for measurement with visual adjustment and 0.9% and 16.2% for measurement without visual adjustment, respectively.

## Discussion

AST belongs to the main tasks of clinical microbiology and laboratories are responsible for providing reliable and comparable results. The most important prerequisite for ensuring correct methodology is the availability of international guidelines, e.g. EUCAST, to which the staff should strictly adhere. However, the reading of disk diffusion results might vary depending on the investigator and, thus, standardization within the laboratory is an important issue. Automated systems for reading and interpretation of inhibition zones have been suggested for improving quality of AST. In previous studies, such systems have been shown to provide acceptable accuracy with non-fastidious bacteria [5,6,8–11,13], although some authors reported poor performance [14]. In this study, we evaluated the ADAGIO system for fastidious bacteria tested on MH-F agar.

In absolute numbers, most errors in our study were MEs leading to false-resistant categorization of an isolate (S1 Table). The visual on-screen adjustment considerably reduced MEs but only slightly reduced numbers of VMEs and mEs (Table 1). Expressed as percentages according to ISO 20776–2 guideline [12], VMEs predominated over MEs and exceeded the acceptance limit of 3% stated by ISO 20776–2. However, this high VME percentage is apparently due to the low number of resistant isolates in the tested collection, which is characteristic for the included species. According to ISO 20776–2, VME rate is not a very indicative marker to validate a test system in such cases (as the VME rate calculation is based only on the number of resistant isolates). Hence, other criteria should be taken into account. CA of 94.8% after visual adjustment clearly fulfils the criterion of CA ≥90% required by ISO, while CA of 81.4% (without visual adjustment) is not sufficient. We therefore conclude that the ADAGIO system provided acceptable accuracy with fastidious bacteria after visual on-screen verification of inhibition zones. However, only poor performance was achieved with *H*. *influenzae* isolates. Previously, inability of automated measurement to read plates inoculated with *Haemophilus* species due to faint growth has been reported [9].

Performance of automated reading with visual adjustment in this study with fastidious bacteria was in general similar or somewhat inferior to that reported by other authors with non-fastidious microorganisms [9,11,14]. Many studies reported VME and ME as calculated based on total number of isolate-antibiotic combinations tested [6,9,11,14]. Reported in this way, VME and ME rates are consequently lower and constituted in our study 0.8% and 3.0% for measurement with visual adjustment, respectively.

We predominantly observed false-resistant ADAGIO measurements, i.e. a trend towards underestimation of zone diameter results. This might be biased by the generally high antibiotic susceptibility of the included species of fastidious organisms. However, this trend is in line with the data shown by Sánchez *et al*. for non-fastidious bacteria with OSIRIS, a predecessor device from Bio-Rad [8], while being in contrast to that demonstrated by Kolbert et al. with OSIRIS [6] and by Medeiros *et al*. with the Sirscan (i2a, France) system [9]. Hombach *et al*. reported almost equal distribution of higher or lower inhibition zone diameters with Sirscan system compared to manual method, with an exception of lower zone diameters with automated system in *Enterococcus* spp. [5]

Although the time needed for reading was not thoroughly evaluated in this study, it obviously was shorter with the ADAGIO system than with manual measurement. In routine work processes, one might be tempted to quickly “assess” inhibition zones visually without really measuring diameters. This unacceptable reading may be fast, but a proper manual measurement including interpretation and documentation definitely takes more time than reading by an automated system. In the study of Medeiros *et al*., the time of automated reading was more than twice as short as that needed for manual reading by technologists [9]. Similarly, Andrews *et al*. recorded time saving with an automated system [10]. However, Geiss *et al*. reported longer times needed with the older version of the BIOMIC (Giles Scientific Inc., USA) zone reader [14]. Another useful feature of automated readers is a possibility to keep records of QC results and to automatically analyze that documentation. Currently, a lot of efforts are made to develop total laboratory automation systems for microbiology. Integration of automated measurements of inhibition zones for disk diffusion method is an important part of this work [15].

In conclusion, the ADAGIO system allowed for automated reading of disk diffusion testing in fastidious bacteria and, after visual validation of the automated results, yielded good categorical agreement with manual reading.

## Supporting Information

S1 Table Susceptibility of isolates determined by manual reading (standard method) and performance of automated reading of disk diffusion antimicrobial susceptibility testing (without visual adjustment) for fastidious bacteria, compared to manual reading as standard method, n = 144. (PDF)
