# Supplementary material for: Evaluation of an Automated System for Reading and Interpreting Disk Diffusion Antimicrobial Susceptibility Testing of Fastidious Bacteria
Source: PLoS One. 2016 Jul 8;11(7):e0159183. doi: 10.1371/journal.pone.0159183 (PMC4938495; doi:10.1371/journal.pone.0159183)
Supplement: S1 Table — (PDF) [file pone.0159183.s001.pdf]

Evaluation of an Automated System for Reading and Interpreting Disk Diffusion Antimicrobial Susceptibility Testing of Fastidious Bacteria

Evgeny A. Idelevich, Karsten Becker, Janne Schmitz, Dennis Knaack, Georg Peters, Robin Köck

S1 Table. Susceptibility of isolates determined by manual reading (standard method) and performance of automated reading of disk diffusion antimicrobial susceptibility testing (**without visual adjustment**) for fastidious bacteria, compared to manual reading as standard method, n=144

Antimicrobial agent	No. of isolate-antibiotic combinations	No. of resistant isolates[a]	No. of susceptible isolates	No. (%) of very major errors[b]	No. (%) of major errors[b]	No. (%) of minor errors[b]	Categorical agreement, %
***S. agalactiae*, n=29**	**348**	**31**	**314**	**3 (9.7)**	**54 (17.2)**	**3 (0.9)**	**82.8**
Penicillin G	29	0	29				
Levofloxacin	29	0	29		4		
Moxifloxacin	29	0	29		8		
Norfloxacin	29	0	29		8		
Teicoplanin	29	0	29		3		

Vancomycin	29	0	29				
Erythromycin	29	7	20	1		2	
Clindamycin	29	4	25	2	6		
Tetracycline	29	20	8		7	1	
Tigecycline	29	0	29		17		
Linezolid	29	0	29		1		
Trimethoprim/ sulfamethoxazole	29	0	29				
***S. dysgalactiae*, n=14**	**168**	**12**	**152**	**1 (8.3)**	**32 (21.1)**	**4 (2.4)**	**78.0**
Penicillin G	14	0	14				
Levofloxacin	14	2	12		2		
Moxifloxacin	14	0	14		6		
Norfloxacin	14	1	13		5		
Teicoplanin	14	0	14		3		
Vancomycin	14	0	14				
Erythromycin	14	2	12	1			

Clindamycin	14	1	13		1		
Tetracycline	14	6	6		5	2	
Tigecycline	14	0	13		8	1	
Linezolid	14	0	14		2		
Trimethoprim/ sulfamethoxazole	14	0	13			1	
***S. pyogenes*, n=25**	**300**	**6**	**293**	**0 (0)**	**69 (23.5)**	**1 (0.3)**	**76.7**
Penicillin G	25	0	25				
Levofloxacin	25	0	25		5		
Moxifloxacin	25	0	25		10		
Norfloxacin	25	2	23		9		
Teicoplanin	25	0	25		8		
Vancomycin	25	0	25				
Erythromycin	25	0	24			1	
Clindamycin	25	0	25		6		
Tetracycline	25	4	21		14		

Tigecycline	25	0	25		16		
Linezolid	25	0	25				
Trimethoprim/ sulfamethoxazole	25	0	25		1		
***S. pneumoniae*, n=28**	**336**	**29**	**301**	**3 (10.3)**	**50 (16.6)**	**5 (1.5)**	**82.7**
Levofloxacin	28	0	28		10		
Moxifloxacin	28	0	28		9		
Norfloxacin	28	1	27	1			
Teicoplanin	28	0	28		6		
Vancomycin	28	0	28		7		
Erythromycin	28	7	21		3		
Clindamycin	28	5	23	1	5		
Tetracycline	28	5	23	1	5		
Linezolid	28	0	28		2		
Trimethoprim/ sulfamethoxazole	28	4	24		2		

Oxacillin	28	7	21				
Cefaclor	28	0	22		1	5	
**Viridans group streptococci, n=18**	**126**	**3**	**123**	**0 (0)**	**1 (0.8)**	**0 (0)**	**99.2**
Penicillin G	18	0	18				
Teicoplanin	18	0	18				
Vancomycin	18	0	18				
Ampicillin	18	0	18				
Cefotaxime	18	0	18				
Cefuroxime iv[c]	18	2	16				
Cefepime	18	1	17		1		
***H. influenzae*, n=13**	**156**	**8**	**134**	**7 (87.5)**	**19 (14.2)**	**9 (5.8)**	**77.6**
Penicillin G	13	2	11	2			
Levofloxacin	13	0	13		5		
Erythromycin	13	0	0			8	

Tetracycline	13	0	13				
Trimethoprim/ sulfamethoxazole	13	1	11	1		1	
Ampicillin	13	1	12				
Amoxicillin/clavulanic acid	13	1	12	1	1		
Cefotaxime	13	0	13		1		
Cefuroxime iv[c]	13	3	10	3	2		
Meropenem	13	0	13				
Ciprofloxacin	13	0	13		2		
Nalidixic acid	13	0	13		8		
***M. catarrhalis*, n=7**	**84**	**1**	**82**	**0 (0)**	**16 (19.5)**	**1 (1.2)**	**79.8**
Levofloxacin	7	0	7				
Moxifloxacin	7	0	7		2		
Erythromycin	7	0	7				
Tetracycline	7	0	7		1		

Trimethoprim/ sulfamethoxazole	7	0	7				
Amoxicillin/clavulanic acid	7	0	7				
Cefotaxime	7	0	7		3		
Cefuroxime iv[c]	7	0	7		2		
Meropenem	7	0	7		5		
Ciprofloxacin	7	0	7				
Nalidixic acid	7	1	6		2		
Cefixime	7	0	6		1	1	
***C. jejuni*, n=10**	**30**	**5**	**25**	**0 (0)**	**10 (40)**	**0 (0)**	**66.7**
Erythromycin	10	0	10		4		
Tetracycline	10	2	8		6		
Ciprofloxacin	10	3	7				
**TOTAL, n=144**	**1548**	**95**	**1424**	**14 (14.7)**	**251 (17.6)**	**23 (1.5)**	**81.4**

[a] Number of results within intermediate category can be calculated by subtracting resistant and susceptible results from the number of isolate-antibiotic combinations tested

[b] Error rates are reported as required by the ISO 20776-2 guideline: VME (%), number of VMEs (i.e. false susceptible results) divided by the number of isolates determined resistant by the standard method; ME (%), number of MEs (i.e. false resistant results) divided by the number of isolates determined susceptible by the standard method); mE (%), number of mEs (i.e. false categorization involving intermediate result) divided by the total number of tested isolates; Categorical agreement (i.e. results within the same interpretative category)

[c] iv, interpretation according to breakpoints for intravenous use
